# Toxic Effect of Fullerene and Its Derivatives upon the Transmembrane β_2_-Adrenergic Receptors

**DOI:** 10.3390/molecules27144562

**Published:** 2022-07-18

**Authors:** Longlong Ren, Zhenxiang Jing, Fei Xia, John Zenghui Zhang, Yang Li

**Affiliations:** 1 College of Mechanical and Electronic Engineering, Shandong Agricultural University, Tai’an 271018, China; renlonglong@sdau.edu.cn (L.R.); lyzhenxiangjing@163.com (Z.J.); 2 NYU-ECNU Center for Computational Chemistry at NYU Shanghai, School of Chemistry and Molecular Engineering, East China Normal University, Shanghai 200062, China; fxia@chem.ecnu.edu.cn (F.X.); zhzhang@phy.ecnu.edu.cn (J.Z.Z.); 3 College of Information Science and Engineering, Shandong Agricultural University, Tai’an 271018, China

**Keywords:** MD simulation, fullerene derivatives, cytotoxicity, β_2_-adrenergic receptor

## Abstract

Numerous experiments have revealed that fullerene (C_60_) and its derivatives can bind to proteins and affect their biological functions. In this study, we explored the interaction between fullerine and the β_2_-adrenergic receptor (β_2_AR). The MD simulation results show that fullerene binds with the extracellular loop 2 (ECL2) and intracellular loop 2 (ICL2) of β_2_AR through hydrophobic interactions and π–π stacking interactions. In the C_60__in1 trajectory, due to the π–π stacking interactions of fullerene molecules with PHE and PRO residues on ICL2, ICL2 completely flipped towards the fullerene direction and the fullerene moved slowly into the lipid membrane. When five fullerene molecules were placed on the extracellular side, they preferred to stack into a stable fullerene cluster (a deformed tetrahedral aggregate), and had almost no effect on the structure of β_2_AR. The hydroxyl groups of fullerene derivatives (C_60_(OH)_X_, X represents the number of hydroxyl groups, X = 4, 8) can form strong hydrogen bonds with the ECL2, helix6, and helix7 of β_2_AR. The hydroxyl groups firmly grasp the β_2_AR receptor like several claws, blocking the binding entry of ligands. The simulation results show that fullerene and fullerene derivatives may have a significant effect on the local structure of β_2_AR, especially the distortion of helix4, but bring about no great changes within the overall structure. It was found that C_60_ did not compete with ligands for binding sites, but blocked the ligands’ entry into the pocket channel. All the above observations suggest that fullerene and its derivatives exhibit certain cytotoxicity.

## 1. Introduction

Since the discovery of fullerene (C_60_), its functions have attracted the intensive interests of scientists [1,2]. Because of the excellent physical and chemical properties of fullerene, it has become one of the most promising nanomaterials, and has been widely used in the pharmaceutical industry [3,4,5,6]. In vivo, fullerene molecules show a tendency to aggregate into nanoparticles which can cross the cell membranes of brains due to their lipophilic properties. Thus, fullerene has been used for drug delivery in vivo because of its high stability and hollow cage-like structure [7,8]. For instance, the diameter of fullerene is comparable to the size of active centers of some viruses, such as HIV, so that it has been utilized to block the entrances of active viral centers and prevent the viruses obtaining nutrition from cells [9,10,11]. Recently, it has been found that nanomaterials composed of fullerene derivatives could stabilize immune effector cells to inhibit the release of proinflammatory mediators, making them potential candidates for treatment of several diseases including asthma, arthritis and multiple sclerosis [12,13,14,15,16].

In addition to the broad applications of fullerene and its derivatives in the pharmaceutical industry, their potential toxicity also drawn the attention of researchers [17,18]. The exposure of largemouth bass to nC_60_ led to lipid peroxidation in the brain and glutathione depletion in their gills, affecting the signal transmission and normal expression of proteins [19,20]. Further experimental studies reported that fullerene nanoparticles destroyed the permeability and integrity of cell membranes as they crossed them [21,22,23,24]. Cell membranes are composed of various lipids and transmembrane proteins responsible for the functions and activities of cells. When the fullerene nanoparticles cross the cell membranes, they not only affect the structures and functions of the lipid membranes, but also the transmembrane proteins. Previous experimental studies also revealed that fullerenes could affect the activity and function of proteins, including potassium channel proteins [25,26], HIV proteases [9,10,11,27,28], serum albumins [29,30,31] and glutathione *S*-transferases [32,33,34].

Using microsecond molecular dynamics (MD) simulations, Monticelli et al. investigated the interaction of C_70_ fullerene with Kv1.2 potassium channel proteins. Their simulated results revealed that the fullerenes acted as blockers to change the conformations and functions of the secondary structures of Kv1.2 [35]. Further MD simulations of fullerenes and their related derivatives indicated that they could bind to hydrophobic sites on the surfaces of proteins through specific interactions such as π–π stacking [36,37]. It is generally understood that such kind of π–π stacking interactions exist between the conjugated surface of C_60_ and the hydrophobic residues of PHE, TRP, and TYR with aromatic rings.

Fullerene and its derivatives worked as competitors to the ligands of target proteins. Kraszewski et al. found that extracellular fullerene molecules blocked the entry of potassium channel proteins and prevented potassium ions from entering the channel. The intracellular fullerene molecules could enter the channel from the side of the cytoplasm and become stuck in the cavity formed by a few helices of channel protein [25,35]. A series of experimental studies about the cytotoxicity of fullerene derivative C_60_(OH)_n_ have also been reported [38,39,40]. Although there has been a great deal of evidence for the toxicity of fullerene and its derivatives, the atomistic interaction details of fullerenes and related proteins remain unclear so far.

**Figure 1:**
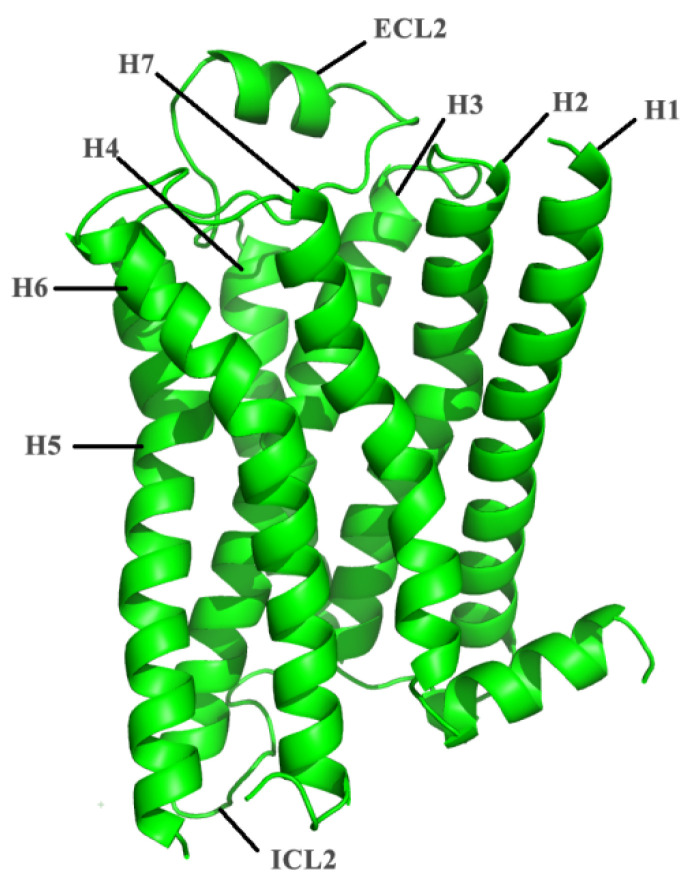
The crystal structure of β_2_AR (PDB ID: 2RH1) and the seven transmembrane helices H1–H7, as well as the ECL2 and ICL2 loops.

In this study, we performed MD simulation to investigate the toxic effect of fullerenes and their hydroxyl derivatives on the β_2_-adrenergic receptor (β_2_AR), which serves as an important target for cardiac and asthma drugs, and is an extensively studied model system within the G-protein coupled receptor (GPCR). β_2_AR is the protein that mediates muscle relaxation in bronchi, as well as vasoconstriction and vasodilation, the structure of which is shown in Figure 1. It has been found that fullerene derivatives are able to suppress disease onset and to reverse established disease in murine asthma models by decreasing airway inflammation and bronchoconstriction. Therefore, we speculate that fullerene molecules can interact with β_2_AR for the treatment of asthma with associated inflammation. Moreover, Yamawaki et al [41] found that the derivative C_60_(OH)_24_ caused cytotoxic injury or cell death in vascular endothelial cells, indicating that exposure to fullerene could represent a risk for atherosclerosis and ischemic heart disease. Furthermore, Wang et al [42]. found that the ECL2 (extracellular loop 2) and the transmembrane helix 7 (TM7) in β_2_AR formed a hydrophobic bridge upon the entry site of the ligand. Moreover, the extracellular top of the binding pocket was shown to be composed of rich hydrophobic residues such as PHE, TYR and LYS. Inspired by the structural features of β_2_AR, we postulate that C_60_ molecules could interact with β_2_AR through hydrophobic interaction. The ligands of β_2_AR are mainly bound by polar hydrogen bonds, so the polar fullerene derivatives may also interact with the β_2_AR. Therefore, it is necessary to study the interaction between β_2_AR and fullerene or its hydroxyl derivatives.

Due to the complexity of the biosystem, it is not practical to investigate experimentally the effects of fullerenes and fullerene derivatives upon β_2_AR. Computer simulation can be a good alternative. Although there have been large quantities of results describing the interactions between C_60_ and proteins observed by experimental and computational methods, no research has been reported focusing on the interaction between C_60_ and β_2_AR. In this work, we used MD simulation to investigate the interactions of fullerene and its hydroxyl derivatives with β_2_AR, to elucidate their toxic effect on the function of β_2_AR based on the simulated trajectories. We mainly aimed to address the following issues: (1) identification of the binding sites of C_60_, C_60_(OH)_4_, and C_60_(OH)_8_ in β_2_AR; (2) the effects of mono-C_60_, five C_60_s, C_60_(OH)_4_, and C_60_(OH)_8_ on the structure of β_2_AR; (3) whether or not C_60_ and C_60_(OH)_X_ (X = 4, 8) compete with the binding site of β_2_AR; (4) whether C_60_ and C_60_(OH)_X_ (X = 4, 8) block protein entry.

## 2. Simulation Details

### 2.1. Construction of Fullerene and Derivative Models

**Figure 2:**
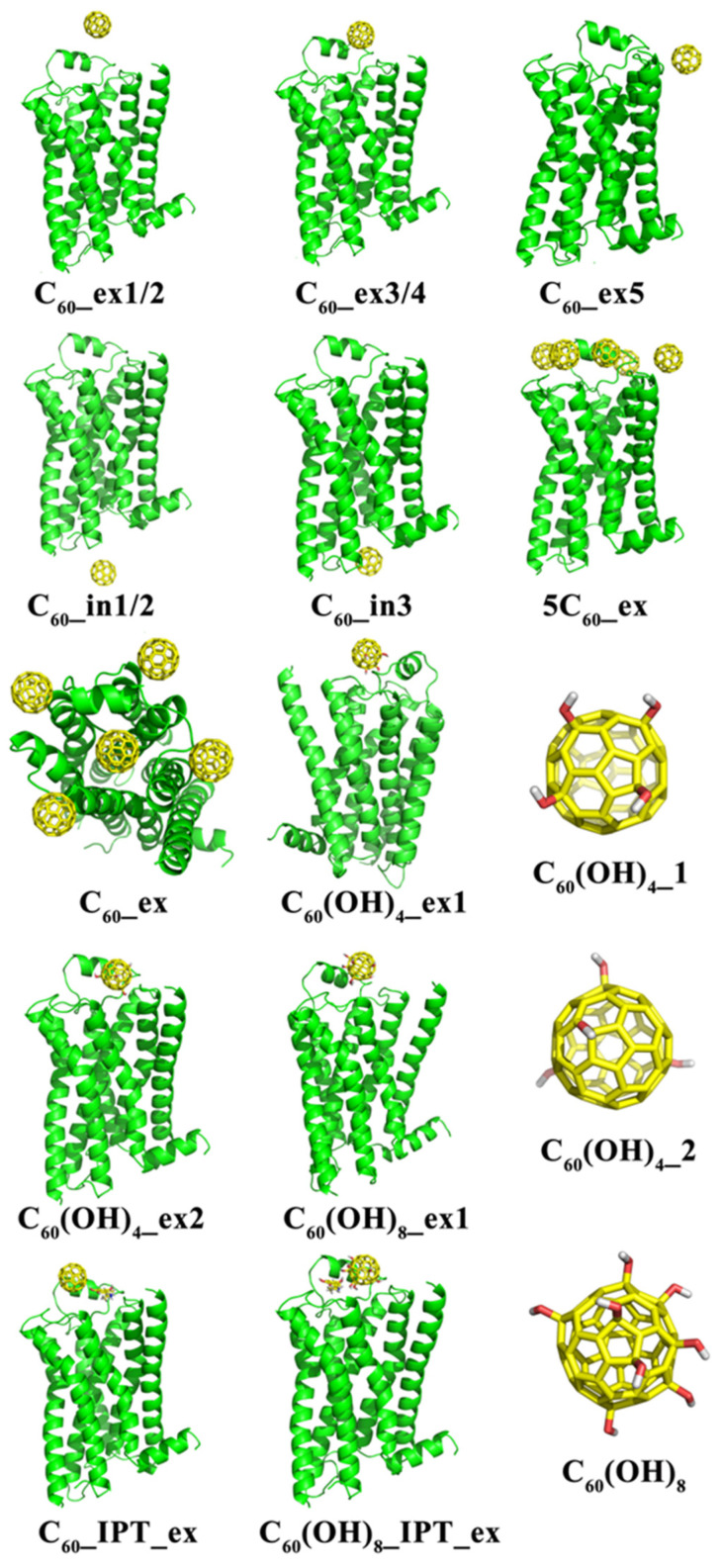
The initial structures of the fullerene derivatives C_60_(OH)_4__1, C_60_(OH)_4__2 and C_60_(OH)_8_ for simulation.

The initial structures of C_60_ fullerene (with radius 3.48 Å) and its hydroxyl derivatives C_60_(OH)_X_ (X = 4 or 8) were built as shown in Figure 2, where X means the number of hydroxyl groups. For the derivative C_60_(OH)_4_ with four hydroxyls, we constructed two representative models C_60_(OH)_4__1 and C_60_(OH)_4__2, with their hydroxyl groups located at different surface positions of C_60_. In the model C_60_(OH)_4__1, the four hydroxyls were located at the close positions of the fullerene spherical surface, with two or three carbon atoms between the two adjacent hydroxyls. In the other model C_60_(OH)_4__2, the four hydroxyls were evenly distributed spherical fullerenes. For the C_60_(OH)_8_ model, there were four hydroxyls close to each other, but the other four hydroxyls were far from each of these four hydroxyls. The structures of fullerene and derivatives were optimized using the B3LYP/6-31G* method in the Gaussian09 software [43]. Then, frequency analysis was carried out to obtain the vibrational force constants for all the bonds and angles involved in the fullerene and derivatives. The RESP charges of all atoms in the fullerenes and derivatives were calculated by fitting the molecular electrostatic potential [44,45]. The van der Waals interactions of pairwise atoms were described using the classical Lennard-Jones potential. The parameters of the cross section *s* and well depth *ε* in the Lennard-Jones potential were taken from the Amber03 force field, with values of 0.34 nm and 0.36 kJ/mol respectively.

### 2.2. Simulation Systems

The initial structure of β_2_AR was extracted from the crystal structure (PDB ID: 2RH1) deposited in the online Protein Data Bank [46]. The crystal structure of β_2_AR contains an endogenous ligand (−)-Isoproterenol [47,48], which is a beta adrenoreceptor agonist used for the treatment of bradycardia (slow heart rate), heart block, and asthma. The (−)-Isoproterenol in the system is abbreviated as ‘IPT’.

To explore systematically the interaction of the β_2_AR protein with the fullerene and derivatives, we constructed fifteen systems composed of β_2_AR protein complexes, DOPC lipids and water molecules. Details of the components in these complex systems are shown in Table 1.

In these complex systems, the initial positions of fullerenes and derivatives were randomly placed at the extracellular or intracellular sides of lipid membranes, indicated by “ex” or “in” in the notation. Initially, to reduce the interaction between the fullerene molecule or fullerene derivatives and β_2_AR, they were spatially separated at distances (defined as the distance between the center of fullerene or derivative’s mass and the β_2_-adrenergic receptor surface) of 1.4 nm for C_60__ex1 (C_60__ex2), 0.7 nm for C_60__ex3 (C_60__ex4), 0.9 nm in C_60__in1 (C_60__in2), 1.0 nm for C_60__IPT_ex, and 0.8 nm for C_60_(OH)_8__IPT_ex. In the simulation of C_60__ex5, the fullerene was in the middle of the ECL1 of β_2_AR and the neighboring lipid membranes. The centroid distance between the fullerene and the lipid membrane corresponded to the membrane thickness at the position of the lipid membrane surface. The fullerene of C_60__in3 was put on the top of the pocket using the helix3, helix4, helix6, and helix7. The IPT of the system C_60__IPT_ex, C_60_(OH)_8__IPT_ex was manually placed at the top of the binding pocket. The specific positions of C_60_, C_60_(OH)_4_, and C_60_(OH)_8_ IPT molecules in the corresponding systems are shown in Figure 2.

### 2.3. Simulation Details

The MD simulations for all systems were carried out using the Amber03 force field in the Gromacs 4.4.5 software [49]. In the simulation, the bonds involving hydrogen atoms were constrained using the LINCS protocol [50] and the non-bonded interactions were updated every five integration steps. The DOPC bilayers, fullerenes, β_2_Ars, and water molecules were coupled to the Berendsen thermostat [51] at 300 K with a relaxation time of 0.1 ps. The surface tension of bilayer liquids was maintained at a magnitude of 440 bar/nm. The pressure perpendicular to the liquid surface was kept at 1.0 bar using Berendsen pressure coupling with a relaxation time of 1.0 ps and a water compressibility of 4.5 × 10^−5^ bar^−1^. The electrostatic interaction of pairwise atoms was estimated using the Particle Mesh Ewald method, and van der Waals interaction within a cutoff 1.2 nm was taken into account. All the systems were first energy-minimized, then heated to 300 K and equilibrated for 300 ns in the NPT ensembles. All data in figures were analyzed based on the equilibrated trajectories of 300 ns MD simulations.

## 3. Result and Discussion

### 3.1. Binding Sites of Monomeric Fullerenes upon *β_2_AR*

**Figure 3:**
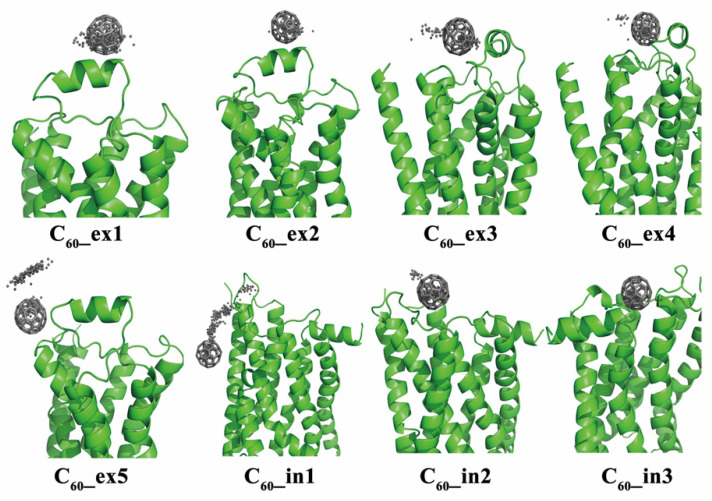
The initial binding sites of monomeric fullerenes in different systems of protein complexes. The black dots represent the center of mass of C_60_ along the MD trajectory sampled every 1 ns and the stick is the ending position of C_60_.

**Figure 4:**
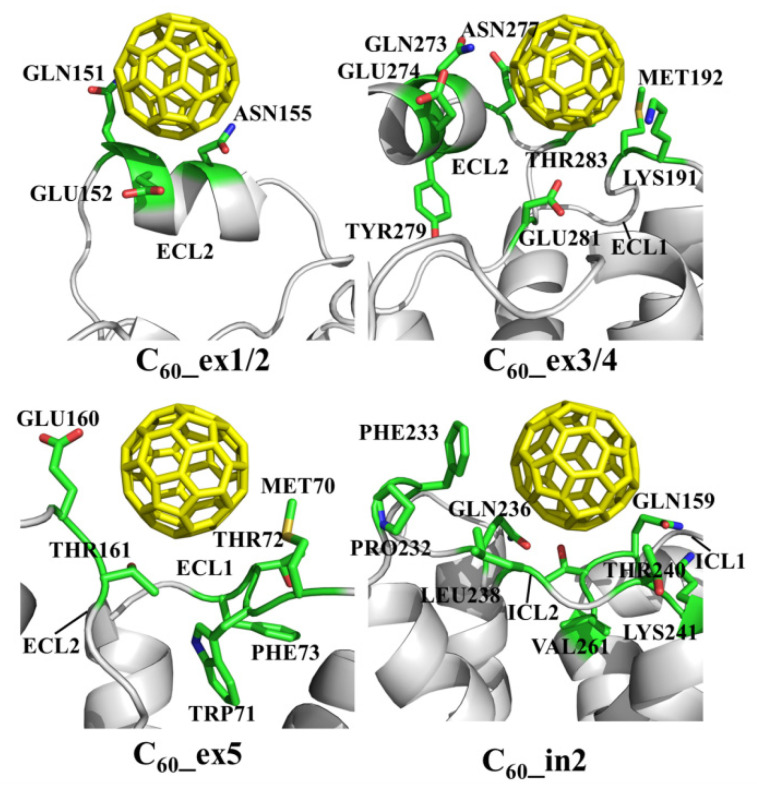
Snapshots of the C_60__ex1/2, C_60__ex3/4, C_60__ex5 and C_60__in2 systems show that the key amino acids of the ECL1 and ECL2 interact with the monomeric fullerenes at the extracellular and intracellular sides.

Figure 3 shows the various binding patterns of monomeric fullerenes to the β_2_AR proteins. The C_60_ molecules in these plots display the final positions in snapshots extracted from MD trajectories. It can be clearly seen that the binding sites of fullerenes were mainly located near the ECL2 or ICL2, except for the system C_60__in1. During the MD simulations, the positions of fullerenes in C_60__ex1/2/3/4/5 and C_60__in2/3 showed almost no large changes compared to their initial positions. The reason for this might be the fact that the β_2_AR is a polar receptor, so it prevents the nonpolar C_60_ from entering. As shown in the MD simulations, the C_60_ molecules in the aqueous phase attached quickly to β_2_ARs within 5 ns. Since the binding pockets of β_2_AR are hydrophilic, the C_60_ molecules prefer to bind to the exposed ECL2 instead of entering it. Figure 4 shows the structural details of the specific amino acids at the binding sites of β_2_AR in C_60__ex1/2, C_60__ex3/4, C_60__ex5 and C_60__in2 interacting with C_60_. It can be seen that the C_60_ molecules were embedded in the cavities formed by the hydrophobic moieties of polar and non-polar amino acids such as GLN, MET, and PHE of ECL2. The aromatic rings of the TYR residues in C_60__ex3/4, PHE in C_60__ex5, and PHE of ICL2 in C_60__in2 formed the π–π stacking interaction to stabilize the conformations of C_60_.

**Figure 5:**
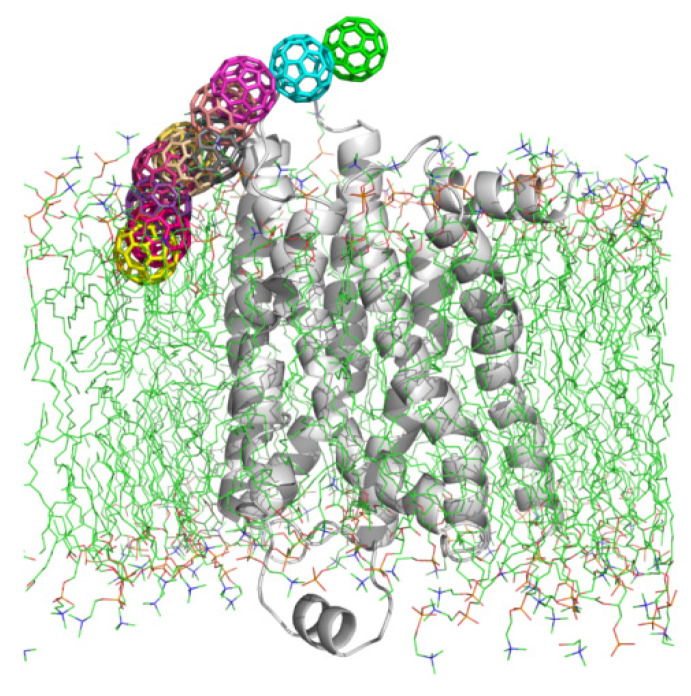
The traced positions of C_60_ extracted from the snapshots of simulated trajectory of the C_60__in1 system. The green and yellow models represent the initial and final positions, respectively.

In Figure 3, it can be observed that the C_60_ in the C_60__in1 simulation eventually entered the lipid membrane, which differed from the C_60__in2 simulation with the same initial structure. To gain insight into the dynamic process of C_60_ entry into the membrane, we extracted a series of snapshots from the MD trajectory and marked the positions of C_60_ at different simulation times using different colors, as shown in Figure 5. The illustration shows that C_60_ gradually squeezed into the lipid membrane during the 300 ns simulation.

**Figure 6:**
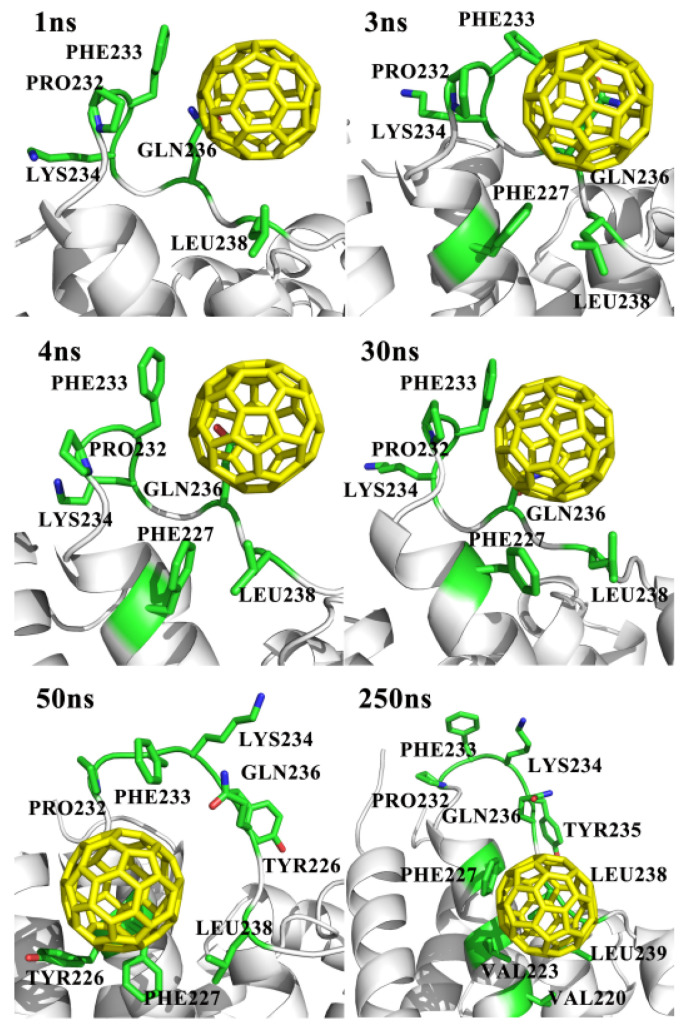
Snapshots of the system C_60__in1 at different simulation times show that the key amino acids such as the PRO232, PHE233, LYS234, GLN236 and LEU238 interacted with the monomeric fullerenes at the extracellular and intracellular sides.

Figure 6 shows the positions of fullerenes in the snapshots extracted from the MD trajectory, with the specific residues surrounded. In the snapshots at 1 ns and 30 ns, the C_60_ molecules showed strong packing interaction with the aromatic rings of PRO and PHE residues. Subsequently, the C_60_ molecules gradually moved away from the ICL2 region, and embedded deeply to interact with the hydrophobic residues in helix4, such the PHE and VAL residues. It can be seen that the loop in the ICL2 region completely flipped after 50 ns, so that the interaction became weak. Finally, the C_60_ molecule was bound to the hydrophobic PHE, TYR, and VAL residues in the helix4, and its conformation changed remarkably.

### 3.2. Binding Sites of Multiple Fullerenes upon *β_2_AR*

**Figure 7:**
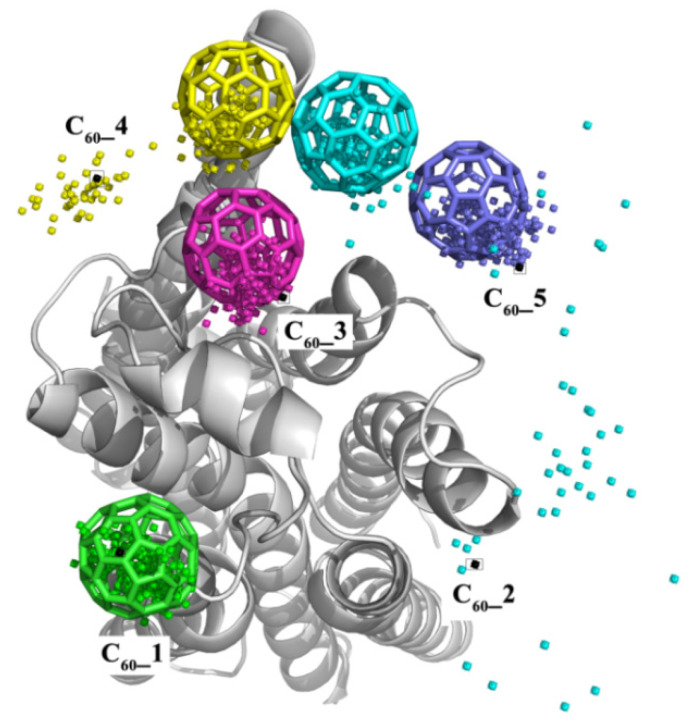
The trajectory of five fullerene molecules in the 5C_60__ex system (top view); different colors represent different fullerene molecules. Each dot represents the average position (center of mass) of C_60_ along the MD trajectory, sampled every 1 ns. The black dots represent the initial positions and the colored stick is the final position of C_60_.

Using a high concentration of fullerenes in experiments, multiple C_60_ molecules should be present to interact with the β_2_AR protein. To explore the interaction mechanism, we randomly placed five C_60_ molecules at the extracellular side of β_2_AR at the beginning of the MD simulation. The separation between C_60_ molecules was beyond 15 Å, and the distances between the centroids of C_60_ and the protein surface was about 7 Å. Figure 7 shows the traces of C_60_ molecules as well as the final stable conformations. The traced dots indicate that although the initial structures of C_60_ were separated far apart from each other, four of them, i.e., C_60__2, C_60__3, C_60__4 and C_60__5, finally tended to form a C_60_ cluster. In particular, the cyan dots reveal that the C_60__2 moved nearly 25 Å from its initial position and finally formed a stable cluster with other C_60_ molecules. C_60__1 could not move around and was trapped in a local stable conformation due to the obstacle of ECL2.

The simulation result shown in Figure 7 indicates that the multiple C_60_ molecules tended to form aggregates at the surface of β_2_AR because of their strong hydrophobic effect. Thus, the hydrophobic surface of C_60_ preferred to interact with the hydrophobic surface of β_2_AR, so that the entropy of the whole protein–protein interaction was most favorable. Furthermore, the simulation revealed that the formed C_60_ cluster remained at the entrance of the β_2_AR binding site, meaning that the high concentration of C_60_ molecules might block the ligand binding to β_2_AR and interrupt the function of β_2_AR.

### 3.3. Binding Sites of Fullerene Derivatives upon *β_2_AR*

**Figure 8:**
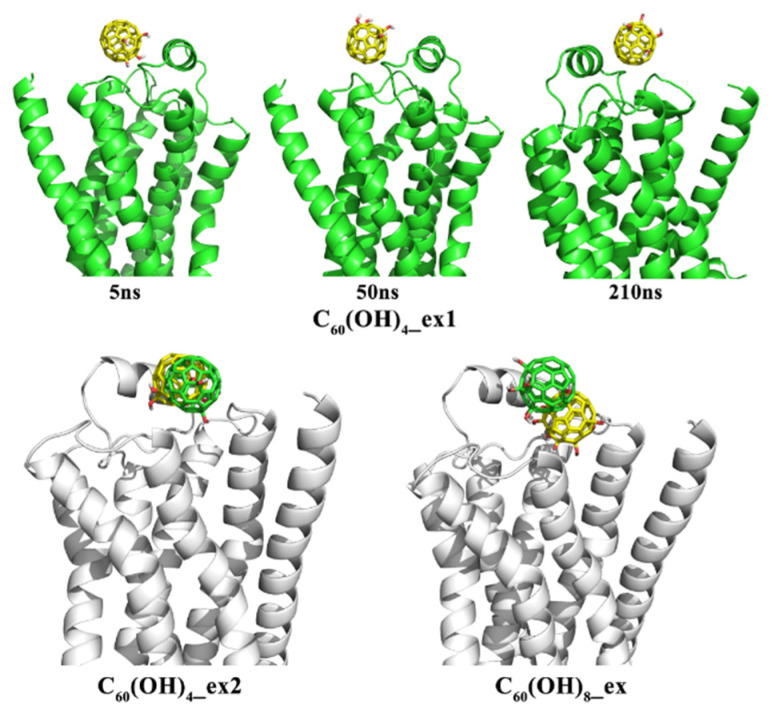
Three snapshots of C_60_(OH)_4__ex1 at different simulation times are shown above, and the two systems C_60_(OH)_4__ex2 and C_60_(OH)_8_ are shown below. The green and yellow models of C_60_(OH)_X_ (X = 4, 8) represent initial and final positions, respectively.

Because C_60_ is nonpolar, the major interaction of fullerene with β_2_AR is hydrophobic. This does not hold true for the fullerene derivatives, since the derivatives C_60_(OH)_X_ (X = 4 or 8) possess polar hydroxyl groups on their surfaces. Figure 8 shows the stable interaction patterns of C_60_(OH)_4__ex1, C_60_(OH)_4__ex2, and C_60_(OH)_8_ with the β_2_AR proteins. It appears that the binding capacity of fullerene derivatives was stronger than that of C_60_ molecules. For example, the hydroxyl groups in C_60_(OH)_4__ex1 formed hydrogen bonds with the GLU and ASN residues in ECL2. In C_60_(OH)_4__ex2, the hydroxyl groups formed hydrogen bonds with the ASP, GLU, and THR residues, and in C_60_(OH)_8__ex, the hydroxyl groups interacted with the HIE, LYS, ASP, and CYX residues. The hydroxyl groups in the fullerene derivatives acted like claws and firmly hooked β_2_AR so that the derivatives blocked the entrance of the β_2_AR binding channels.

Although C_60_(OH)_4__ex1 and C_60_(OH)_4__ex2 each contain four hydroxyl groups, their interaction patterns with β_2_AR were not entirely similar. In the first 40 ns, the four hydroxyl groups in C_60_(OH)_4__ex1 formed hydrogen bonds with β_2_AR. After 50 ns, the hydrogen bonds between C_60_(OH)_4__ex1 and β_2_AR were broken and the hydroxyl groups pointed toward the aqueous phase. This phenomenon was not observed in the simulation of C_60_(OH)_4__ex2, in which the four hydroxyl groups were located far away from each other. By analyzing the interaction between C_60_(OH)_4__ex2 and β_2_AR, we found that the strong hydrogen bonds formed between C_60_(OH)_4__ex2 and the ASP164 and GLU152 residues in ECL2 were retained throughout the 300 ns simulation, which maintained the stable conformation of C_60_(OH)_4__ex2. Comparison of the MD simulations of C_60_(OH)_4__ex1 and C_60_(OH)_4__ex2 revealed that the position of hydroxyl groups had a great influence on the binding capacity of fullerene derivatives. When the hydroxyl groups on the surface of C_60_ stayed close to each other, stable hydrogen bond interaction with β_2_AR was difficult to maintain, due to the steric effect. 

Meanwhile, the number of hydroxyl groups might affect the binding capacity of derivatives to β_2_AR. In order to explore the dependence of binding capacity on the number of hydroxyl groups, we also carried out MD simulation for the C_60_(OH)_8__ex system, as shown in Figure 8. This showed that the C_60_(OH)_8_ molecule was deeply embedded in the β_2_AR. The hydroxyl groups behaved like several claws firmly grasping the β_2_AR, blocking the binding entry of ligands. Strong hydrogen bonds formed between the hydroxyl groups and each of the residues ASP164 and GLU152, and were not broken during the 300 ns simulation time. The difference between C_60_(OH)_4__ex2 and C_60_(OH)_8__ex indicates that increasing the number of hydroxyl groups in fullerenes could enhance their ability to bind with β_2_AR.

### 3.4. Stability of *β_2_AR* with Binding

**Figure 9:**
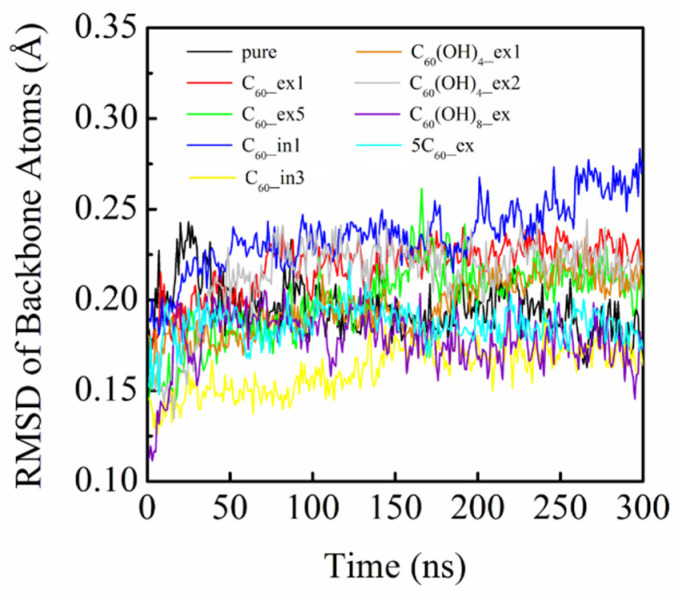
The calculated RMSD curves of the entire protein backbones with respect to the β_2_AR crystal structure.

To explore the effect of fullerene binding, and that of its derivatives, on the stability of β_2_AR, we calculated the RMSDs of β_2_AR backbones for the eight systems, as shown in Figure 9. The RMSDs of all curves, except the C_60__in1 system, remained around 0.2 nm in the simulations, consistent with the results simulated by Romo et al [52]. The RMSD values fluctuated around 0.2 nm, indicating that the cores of β_2_AR were extremely rigid and less susceptible to external binding with C_60_ and its derivatives. For the system C_60__in1, the RMSD reached 0.27 nm at 300 ns, a little higher than that of pure β_2_AR. The large RMSD change exhibited by β_2_AR in C_60__in1 was caused by the hydrophobic interaction of the ICL2 and helix3 with C_60_. In contrast, the RMSD values of C_60__in3 were relatively lower than those of the pure β_2_AR system. The reason for hislies in the fact that the fullerene molecule in C_60__in3 was trapped in the cavity formed by helix 3, helix 4, helix 6 and helix 7, which hindered the cooperative movement of helices in β_2_AR. Previous studies reported that β_2_AR could be activated by the cooperative movement of seven helices, and the ligand binding relied on the collaborative movement of seven helices [53,54,55,56].

**Figure 10:**
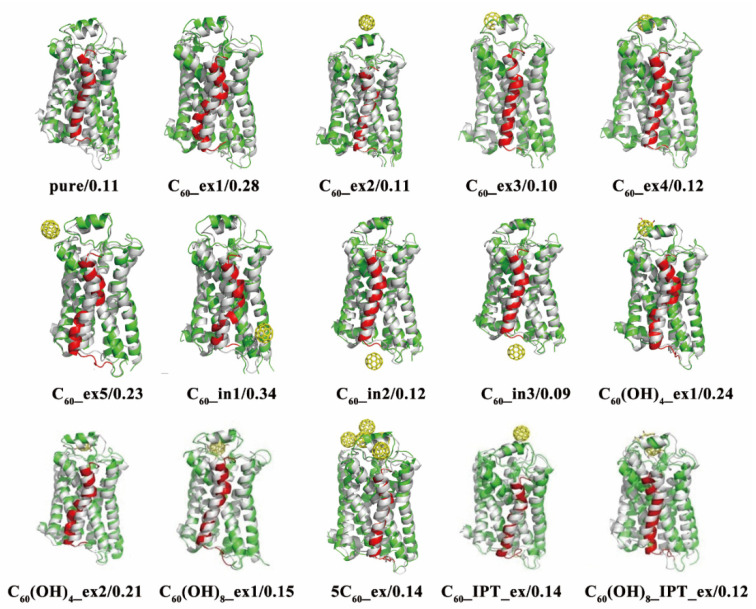
The conformational changes of the simulated systems. The gray parts of the illustrations denote the crystal structures, and the last snapshots in the simulated system trajectories are shown in green, with the helix4 of each highlighted in red. The yellow models represent C_60_ and C_60_(OH)_X_ molecules, and the RMSD values of helix4 with respect to the crystal structure are listed below the structures.

**Figure 11:**
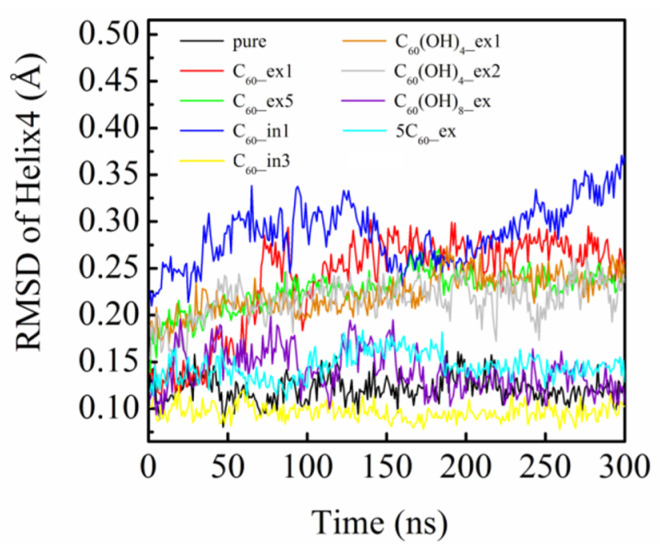
The calculated RMSD curves of helix4 in nine systems with respect to the β_2_AR crystal structure.

We further analyzed the RMSD change of secondary structures of β_2_AR, especially for the key helix4. Figure 10 clearly shows the average structural changes of helix4 in each system, with respect to the crystal structure as a reference. It can be observed that the helix4 in the pure β_2_AR showed no remarkable structural change, while those in C_60__ex2, C_60__ex3, C_60__ex4, C_60__in2, C_60__in3, C_60_(OH)_8__ex, and 5C_60__ex changed little. However, the helices in C_60__ex1, C_60__ex5, C_60__in1, C_60_(OH)_4__ex1, and C_60_(OH)_4__ex2 were severely distorted from the crystal structures. Figure 11 shows the RMSD values of helices 4 calculated for the nine systems in Figure 9. The RMSD change of helix4 for C_60_(OH)_4__ex1/2 was larger than that of C_60_(OH)_8__ex. This could be attributed to the strong hydrogen bonding interaction between C_60_(OH)_8_ and β_2_AR in C_60_(OH)_8__ex. In addition, the C_60_ cluster formed in 5C_60__ex had no substantial influence on the stability of helix 4. This suggests that increasing the number of C_60_ molecules has minimal influence on the stability of β_2_AR.

**Figure 12:**
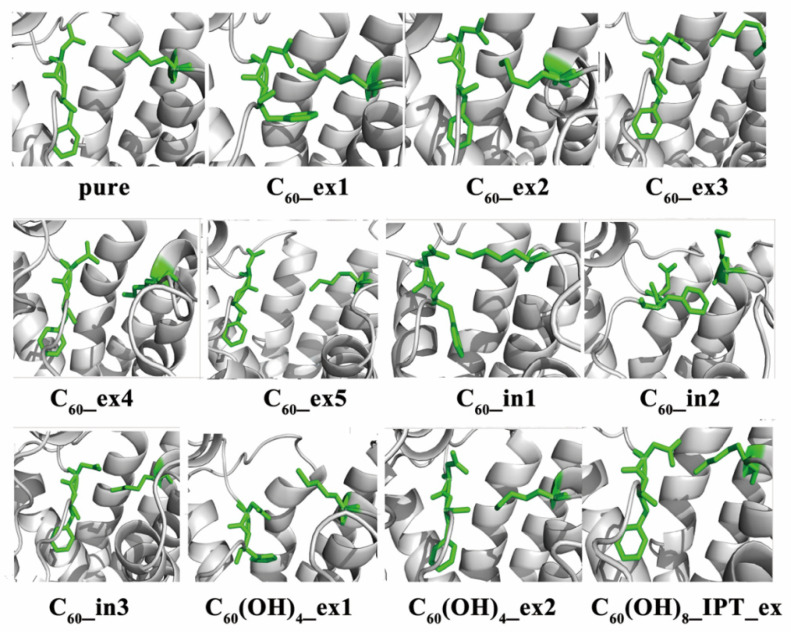
The conformations of PHE193 in snapshots at the final 1 ns.

To uncover the mechanism of the helix4 structural change, we analyzed the conformation evolution of β_2_AR, as shown in Figure 12. We found that it was closely associated with the conformational change of the key residue PHE193 in ECL2. The PHE193 showed an obvious flip in C_60__ex1, C_60__in1, and C_60_(OH)_4__ex1. Compared to the RMSD curves in Figure 11, we found that these three systems had large RMSD values for the fluctuation of helix4. This is because the fullerene molecules outside the membrane underwent hydrophobic and π–π stacking interaction with PHE193, and the fullerene molecules in the membrane mainly affected the structure of helix4 by changing the structure of ICL2. Therefore, it was postulated that the structural changes of helix4 would be directly related to the structural changes of PHE193.

### 3.5. Competitive Mechanism of C_60_/C_60_(OH)_8_ and Isoproterenol

**Figure 13:**
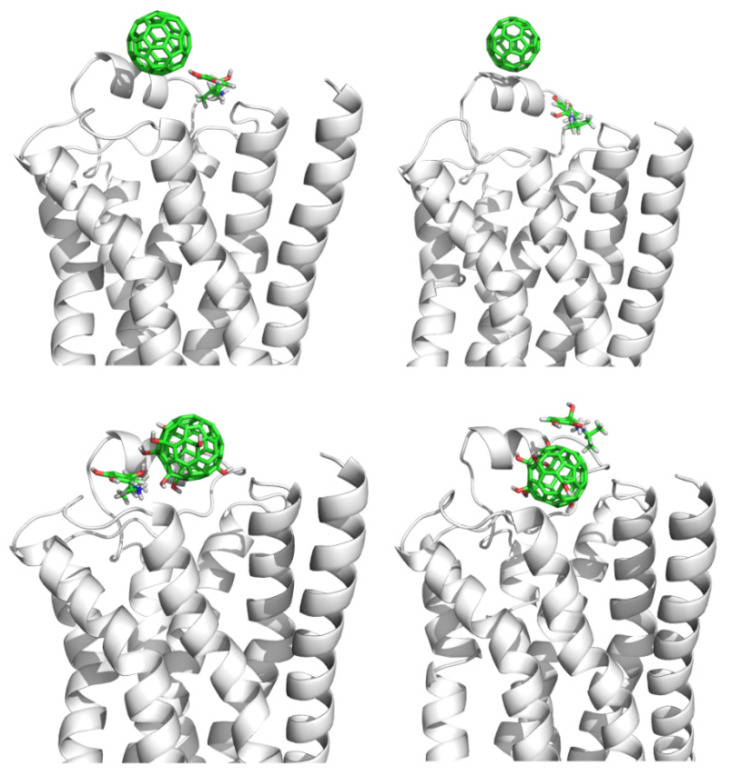
The initial (**left**) and final (**right**) structures of the C_60__IPT_ex (**upper**) and C_60_(OH)_8__IPT_ex (**lower**) systems. The C_60_, C_60_(OH)_8_, and isoproterenols are colored green and the β_2_ARs are grey.

Previous experiments have shown that fullerene molecules and fullerene derivatives may compete with ligands for protein binding sites. In our study, we built two systems, labeled as C_60__IPT_ex and C_60_(OH)_8__IPT_ex, to investigate the competitive mechanisms of C_60_ and C_60_(OH)_8_. Here ‘IPT’ is used as the abbreviated name for isoproterenol. Although, the C_60_ molecule and isoproterenol were both placed above the binding site, we found that C_60_ acted as a single fullerene and had no influence on the isoproterenol within the simulation time, as shown in Figure 13. This result can easily be understood by the hydrophobic nature of pure C_60_. Then we placed C_60_(OH)_8_ and isoproterenol together, and found that C_60_(OH)_8_ quickly combined with β_2_AR through hydrogen bonds, and the isoproterenol was relegated to the outside. It is interesting that the benzene ring and C_60_(OH)_8_ were packed together. When there were many hydroxyl fullerene derivatives outside the cell membrane, they were likely to compete with ligands for the binding site, thus affecting the signal transduction of β_2_AR to the following proteins.

## 4. Conclusions

In this work, we studied for the first time the interactions between fullerene and fullerene derivatives and β_2_AR, using a series of all-atom MD simulations. We established fifteen systems by placing fullerene and fullerene derivatives at different positions outside and inside the membrane. Our results show that the unmodified fullerene was unable to enter the ligand binding sites, instead mainly binding with the loop of ECL2 and ICL2 on β_2_AR through hydrophobic interactions and π–π stacking interactions, especially with residue PHE193 on ECL2, and PHE and PRO residues on ICL2. In addition to having a strong hydrophobic interaction with nonpolar amino acid residues, fullerene molecules also underwent obvious hydrophobic interaction with hydrophobic carbon atoms on polar amino acids, such as GLN, GLU, ASP, LYS, TYR, and THR. In most cases (except C_60__in1), fullerene and derivatives did not enter the lipid membrane, but remained around the β_2_AR. In the C_60__in1 trajectory, due to the π–π stacking interactions of the fullerene molecule with PHE and PRO residues on ICL2, ICL2 completely flipped towards the fullerene direction along with the movement of fullerene slowly into the lipid membrane. Five fullerenes tended to stack into a stable fullerene cluster (a deformed tetrahedral aggregate) rather than interacting alone with β_2_AR. In addition, as the simulation time extended, the position of the fullerene aggregates became almost motionless, just above the receptor binding pocket, which would block the movement of ligands into protein channels and hinder the ligands’ binding to proteins. The binding capacity between polyhydroxy derivatives and β_2_AR was stronger compared with unmodified fullerene molecules.

The hydroxyl groups of fullerene derivatives C_60_(OH)_4_ and C_60_(OH)_8_ can form strong hydrogen bonds with the ECL2, helix6, and helix 7 of 2AR. The hydroxyl groups firmly grasped the β_2_AR receptor like several claws, blocking the binding entry of ligands. The position and number of hydroxyl groups on fullerene derivatives also seriously affected the interaction between the two groups. The more dispersed the hydroxyl position and the greater the hydroxyl number, the stronger was the binding activity with the β_2_AR.

We further explored the effect of fullerene and its derivatives on β_2_AR structure. The results indicated that fullerenes and their derivatives did not have an obvious impact on the whole structure of the receptor, but could affect the structure of helix4 in some systems, especially in the systems C_60__ex1, C_60__ex5, C_60__in1, C_60__in3, C_60_(OH)_4__ex1, and C_60_(OH)_4__ex2. The helix4 structures in the above systems were seriously distorted, with the upper part twisted to the right, and the lower part distorted to the left. Moreover, the more hydroxyl in the fullerene derivative, the smaller was the effect on the β_2_AR structure.

In summary, fullerenes and fullerene derivatives can interact with β_2_AR by hydrophobic interactions, π–π stacking interactions and hydrogen bonding. This combination may block the entry of ligands into protein binding sites, and also may compete with ligands for binding sites, thereby affecting the normal biological function of β_2_AR. Therefore, in future medical research it is important to consider the effect of fullerene and its derivatives on β_2_AR, to further reduce its toxicity to biological cells.

## Figures and Tables

**Table 1:** Details of the constructed complex systems composed of fullerene, fullerene derivatives and β_2_AR, with the 122 DOPC lipids and different numbers of water molecules.

Notations	Components	Number of DOPCs/Waters
Pure_β_2_AR	Without fullerene or fullerene derivative	122/12274
C_60__ex1, C_60__ex2	One C_60_ in the extracellular side	122/12274
C_60__ex3, C_60__ex4	One C_60_ in the extracellular side	122/12202
C_60__ex5	One C_60_ in the extracellular side	122/12113
C_60__in1, C_60__in2	One C_60_ in the intracellular side	122/12035
C_60__in3	One C_60_ in the intracellular side	122/11542
C_60_(OH)_4__ex1	One C_60_ with 4 OH in the extracellular side	122/11153
C_60_(OH)_4__ex2	One C_60_ with 4 OH in the extracellular side	122/11375
5C_60__ex	Five C_60_s in the extracellular side	122/13899
C_60_(OH)_8__ex1	One C_60_ with 8 OH in the extracellular side	122/12059
C_60__IPT_ex	One C_60_ and one isoproterenol in the extracellular side	122/12308
C_60_(OH)_8__IPT_ex	One C_60_ with 8 OH and one isoproterenol molecule in the extracellular side	122/12902

## Data Availability

The data presented in this study are available on request from the corresponding author. The data are not publicly available due to privacy.
